# Assessment of Bacterial Community Assembly Patterns and Processes in Pig Manure Slurry

**DOI:** 10.1371/journal.pone.0139437

**Published:** 2015-09-30

**Authors:** Priyanka Kumari, Hong L. Choi, Sartika I. A. Sudiarto

**Affiliations:** 1 Department of Agricultural Biotechnology, Research Institute for Agriculture and Life Science, Seoul National University, Gwanak, Seoul, 151-921, Republic of Korea; 2 Resourcification Research Center for Crop-Animal Farming (ReCAF), Gwanak, Seoul, Republic of Korea; Wilfrid Laurier University, CANADA

## Abstract

The bacterial community assembly patterns and processes are poorly understood in pig manure slurry. We collected pig manure slurry samples during the winter and summer seasons from eight commercial pig farms in South Korea. The V3 region of 16S rRNA genes was PCR amplified and sequenced using paired-end Illumina technology for in-depth characterization of bacterial community. *Firmicutes*, *Bacteroidetes*, *Proteobacteria*, *Spirochaetes*, and *Tenericutes* were the predominant bacterial phyla present in slurry samples. Bacterial taxonomic community composition was not influenced by the season; however, phylogenetic community composition was affected by seasonal variations. The community composition and diversity patterns were strongly influenced by pH. The bacterial diversity indices showed a unimodal relationship with pH. Phylogenetic signals were detected over only short phylogenetic distances, revealing that closely related bacterial operational taxonomic units (OTUs) tend to co-occur in the same environment; hence, they are ecologically similar. Across all samples, a niche-based process, through strong environmental filtering imposed by pH, primarily governed bacterial community assembly; however, in samples close to the neutral pH range, the role of environmental filtering was decreased due to neutral community assembly. In summary, pH emerged as the major physico-chemical variable in pig manure slurry that regulates the relative importance of niche-based and neutral processes in shaping the community assembly of bacteria.

## Introduction

The global intensification of pig farming has led to the generation of high levels of pig manure slurry [1]. Pig manure slurry generation within South Korea alone is approximately 20 million tons per year [2]. Pig manure slurry is generally stored in a large outdoor storage tank for several months and then applied to agricultural lands as fertilizer. Although pig manure slurry is an important resource as fertilizer and soil conditioner, the handling of a large volume of slurry samples may cause several problems, such as the emission of odorous compounds and contamination of soil and runoff water with pathogenic microorganisms [3–6]. Furthermore, the extended and disproportionate use of pig manure slurry can cause soil deterioration.

Cultivation-based and classical molecular fingerprinting methods (e.g., DGGE, SSCP, cloning and Sanger sequencing of rRNA genes) are predominantly used to characterize the microbial community of pig manure slurry [7–11]. More recently, high-throughput sequencing methods have also been used to analyze the bacterial community present in fresh pig slurry [12], pig wastewater anaerobic lagoons [13], and pig wastewater treatment plants [14]. However, these studies have primarily investigated the patterns without investigating the processes shaping the community assembly of bacteria in pig manure slurry. Two types of processes, niche-based (deterministic) and neutral (stochastic) processes, influence bacterial community assembly. In niche-based processes, community assembly is delimited by abiotic and biotic factors [15], whereas, in neutral processes, community assembly is governed by probabilistic dispersal and ecological drift [16,17].

In this study, we collected pig manure slurry samples during the winter and summer seasons from eight commercial pig farms in South Korea to address the following objectives: (i) to investigate the effect of seasonal variations and influence of physico-chemical characteristics on bacterial community composition and diversity and (ii) to detect the processes that are important for shaping the community assembly of bacteria.

We used a phylogenetic framework to detect the community assembly processes [18,19], in which phylogenetic community composition is coupled with null models, and deviations from the null model expectancy are used to infer the relative influence of niche-based and neutral processes [17,20,21].

## Materials and Methods

### Sample collection

Slurry samples were collected in the winter (January) and summer (June) of 2013 from eight large and medium sized, privately owned commercial pig farms (2000–6000 pigs) in South Korea. Prior access permissions were obtained from farm owners, and they can be contacted for future permissions. Each sampled farm has a large outdoor storage tank, and slurry samples were collected from the top one-meter depth of the surface of the storage tank. After thorough mixing, one liter of slurry was collected in a sterile plastic bottle from five different points of the tank. The slurry samples were immediately placed in ice and transported to the laboratory (within approximately 4 h) for further analyses.

### Physico-chemical analysis

The specific gravity (SG) and pH of slurry samples were determined using a hydrometer (DH.DKDL06, Daihan Scientific, South Korea) and a portable pH meter (D-82362, WTW, Germany), respectively. Temperature and electrical conductivity (EC) were measured using a digital thermometer (HI-98501, Hanna Instruments, USA) and a conductivity meter (EC-214, Hanna Instruments, Italy), respectively. Oxidation reduction potential (ORP) and dissolved oxygen (DO) were measured using an ORP meter (RM-20P, DKK-TOA, Japan) and a DO meter (YSI-5100, YSI, USA), respectively. Total solid (TS), volatile solid (VS), fixed solid (FS) and biological oxygen demand (BOD_5_) were analyzed according to standard methods [22]. Total chemical oxygen demand (TCOD), soluble chemical oxygen demand (SCOD), total nitrogen (TN), ammoniacal nitrogen (NH_3_-N), nitrate (NO_3_) and total phosphorus (TP) were measured using a DR 5000 UV—vis spectrophotometer (DR-5000, HACH Co., USA). Total organic carbon (TOC) was estimated with a TOC analyzer (Shimadzu 5050A, Japan) at the National Instrumentation Center for Environmental Management (NICEM, South Korea).

### DNA extraction and PCR amplification

A volume of 5 ml of homogenized slurry sample was centrifuged at 14,000 × *g* for 5 min, and the resultant pellet was used for DNA extraction using a PowerSoil DNA isolation kit (MoBio Laboratories, USA). The purified DNA was stored at −20°C until PCR amplification. The V3 region of bacterial 16S rRNA genes was PCR amplified using barcoded primer pairs 338F/533R [23].

### Illumina sequencing and data processing

The amplicons were sequenced at the Beijing Genome Institute (BGI) (Hong Kong, China) using paired-end (2×150 nt) Illumina sequencing with a Hiseq2500 system (Illumina, USA). The standard Illumina library preparation, sequencing and initial quality control were performed as described previously [24]. The mothur software package was used to process the sequence data [25]. First, paired-end sequence assembly was generated using the ‘*make*.*contigs*’ command in mothur prior to quality trimming, sequence filtration and alignment against a SILVA alignment (http://www.arb-silva.de/). Next, the ‘*pre*.*cluster*’ and ‘*chimera*.*uchime*’ commands in mothur were used to remove the sequencing errors and chimeric sequences, respectively [26,27]. Taxonomic annotations of all of the high quality sequences were obtained via ‘*classify*.*seq*’ command in mothur using the EzTaxon-e database (http://eztaxon-e.ezbiocloud.net/) [28]. We used ‘*remove*.*lineage*’ command in mothur to remove mitochondrial, chloroplast, Archaea, Eukarya and unknown sequences. A random subset of 5677 sequences per sample was generated using the ‘*sub*.*sample*’ command in mothur prior to statistical analysis. The bacterial operational taxonomic unit (OTU) matrix was built using ‘*dist*.*seqs*’ command in mothur, and the generated distance matrix was used to cluster sequences into OTUs by mothur’s ‘*cluster*’ command using the average linkage algorithm. Finally, ‘*make*.*shared*’ command was used to generate the bacterial OTUs at a cutoff value of 0.03, and diversity indices were calculated using ‘*summary*.*single*’ command in mothur. The phylogenetic diversity index (Faith’s PD) was also calculated [29]. All the sequence reads used in this study were uploaded to metagenomic-RAST server [30] under MG-RAST IDs 4624519.3–4624534.3.

### Statistical analyses

A paired t-test and the Wilcoxon rank-sum test were used on normal and non-normal data, respectively, to check the effect of the season on the diversity and relative abundance of dominant bacterial phyla. The Bray-Curtis (taxonomic) and weighted UniFrac (Phylogenetic) distance matrices were calculated in vegan and picante R packages [31,32], respectively. The differences in bacterial community composition (both taxonomy and phylogeny-based) were visualized using non-metric multidimensional scaling (NMDS) plots. Analysis of similarity (ANOSIM) was used to test the effect of the season on bacterial community structure (permutations = 999). After removing the highly correlated physico-chemical variables (Spearman's *r* ≥ 0.6), the remaining physico-chemical variables were fitted onto ordination space using the ‘*envfit*’ function of the vegan R package (permutations = 999). The resultant significant variables from environmental fitting analysis were further correlated to the diversity indices and relative abundance of the dominant bacterial phyla using linear and polynomial functions.

Phylogenetic signals were evaluated using a Mantel correlogram with 999 randomizations between the OTU phylogenetic distances and OTU niche distances (i.e., differential environment requirements of each OTUs) by following the procedures described previously [20,33]. Phylogenetic signal in ecological niches suggest that closely related organisms tend to prefer similar habitat than distantly relative organisms [34]. Phylogenetic signals were detected only over short phylogenetic distances (see results); therefore, we used the standardized effect size of the mean nearest taxon distance (SES.MNTD) to quantify phylogenetic relationships between closely related taxa. The SES.MNTD (abundance-unweighted) were calculated using the ‘*ses*.*mntd*’ function of the picante R package. The SES.MNTD generates the differences in the MNTD between the observed and null communities (generated using random shuffling of taxa labels 999 times along the tip of the phylogeny) standardized by the standard deviation of the MNTD in null communities [19]. The significant deviation of the SES.MNTD value greater than zero suggests phylogenetic evenness, whereas deviation less than zero indicates phylogenetic clustering. All the analysis steps performed were repeated after removing singleton OTUs to ensure that these patterns were not driven by rare OTUs.

## Results and Discussion

After correlation analysis, highly correlated variables (Spearman's *r* ≥ 0.6) were removed, and only temperature, pH, TOC, total nitrogen, C/N ratio, total phosphorus, NO_3_, and BOD_5_ were used in further analyses. Temperature, total nitrogen and C/N ratio differed significantly between seasons (*P* < 0.5 in all cases), while pH, TOC, total phosphorus, NO_3_, and BOD_5_ did not vary significantly between seasons (Table 1).

**Table 1:** Physico-chemical characteristics of pig manure slurry samples.

Sample	Site name	Latitude	Longitude	Season	pH	Temp* (°C)	TOC (mg/L)	TN* (mg/L)	CN* ratio	TP (mg/L)	NO_3_ (mg/L)	BOD_5_ (mg/L)
S1	Suwon	N 37 15′ 36.0″	E 126 58′ 48.0″	Summer	6.05	26.9	23570	3390	6.95	1610	1130	1825
S2	Yeoju	N 37 17′ 24.0″	E 127 43′ 12.0″	Summer	7.72	26.8	6270	2645	2.37	920	270	7860
S3	Yeongju	N 36 48′ 36.0″	E 128 40′ 12.0″	Summer	7.69	25.8	2957	1335	2.22	1000	390	7866
S4	Yeongju	N 36 18′ 36.0″	E 128 42′ 00.0″	Summer	7.58	29.1	5265	915	5.75	820	420	5130
S5	Jeongeup	N 35 39′ 00.0″	E 126 51′ 00.0″	Summer	7.29	30.3	23970	2860	8.38	870	510	6300
S6	Dangjin	N 36 49′ 48.0″	E 126 43′ 12.0″	Summer	6.99	31.7	9090	315	28.9	650	980	7940
S7	Gunwi	N 36 13′ 48.0″	E 128 33′ 36.0″	Summer	6.99	29.6	20870	5760	3.62	3170	710	10040
S8	Dangjin	N 36 54′ 00.0″	E 126 41′ 24.0″	Summer	7.07	20.0	13195	4800	2.75	860	920	2740
W1	Suwon	N 37 15′ 36.0″	E 126 58′ 48.0″	Winter	6.27	13.2	39130	14200	2.76	2510	390	438
W2	Yeoju	N 37 17′ 24.0″	E 127 43′ 12.0″	Winter	6.40	10.2	22165	10650	2.08	3200	880	185
W3	Yeongju	N 36 48′ 36.0″	E 128 40′ 12.0″	Winter	8.37	8.40	4847	5050	0.96	2510	740	5430
W4	Yeongju	N 36 18′ 36.0″	E 128 42′ 00.0″	Winter	7.69	11.7	12465	6000	2.08	2170	290	7810
W5	Jeongeup	N 35 39′ 00.0″	E 126 51′ 00.0″	Winter	7.87	11.4	13780	4400	3.13	1000	830	1150
W6	Dangjin	N 36 49′ 48.0″	E 126 43′ 12.0″	Winter	7.39	12.1	22360	11200	2.00	100	910	515
W7	Gunwi	N 36 13′ 48.0″	E 128 33′ 36.0″	Winter	8.96	10.3	7240	2600	2.78	900	180	238
W8	Dangjin	N 36 54′ 00.0″	E 126 41′ 24.0″	Winter	7.40	11.0	16775	6050	2.77	1560	210	9680

*P < 0.05 (variables that were significantly different between seasons).

**Fig 1:**
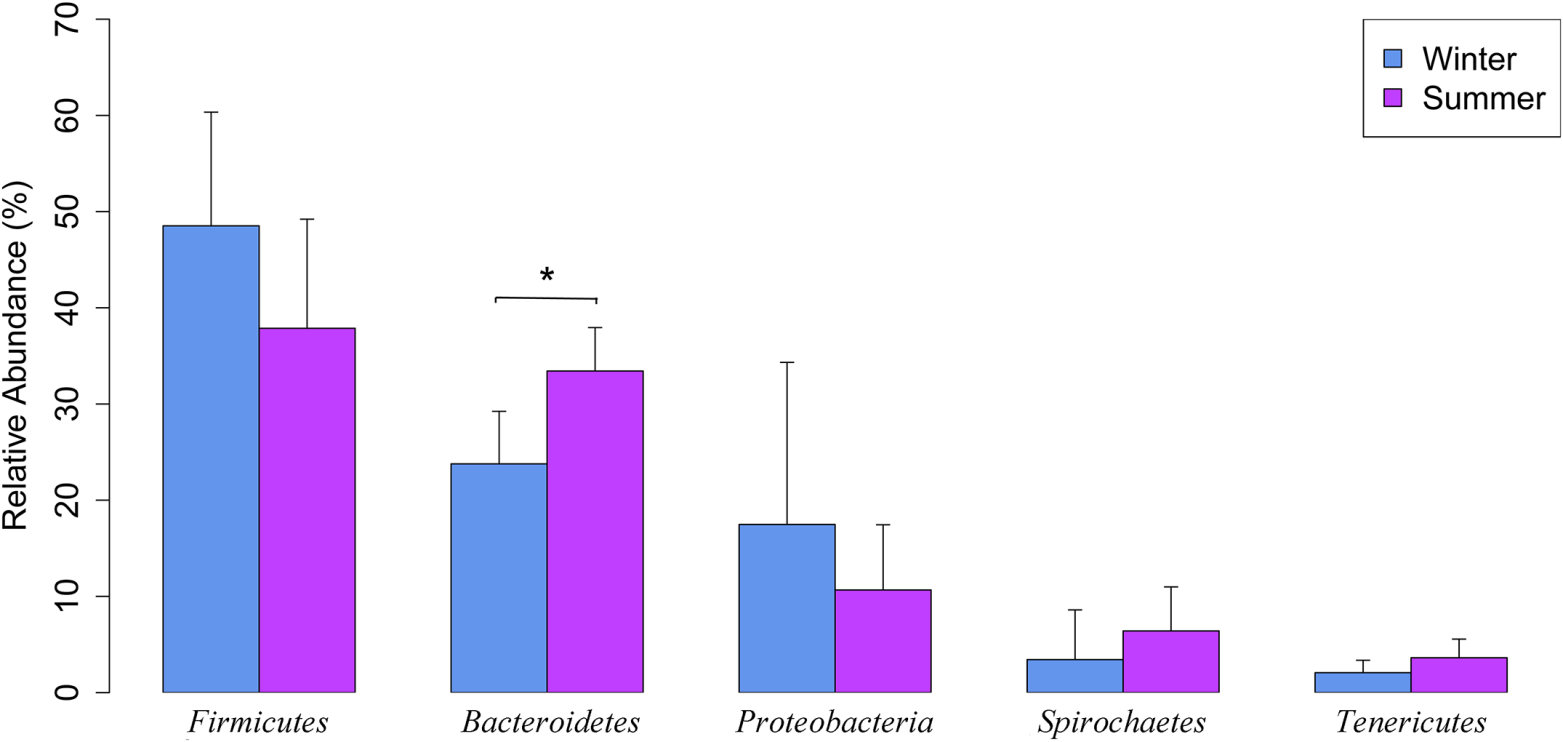
Seasonal variations in the relative abundance (means ± SD) of the most dominant bacterial phyla in pig manure slurry.

We obtained 4,335 OTUs (at a ≥ 97% sequence similarity cut-off) from 96,509 reads (5,677 randomly selected reads per sample). The most abundant phyla detected were *Firmicutes* (42.8%), *Bacteroidetes* (28.5%), *Proteobacteria* (13.1%), *Spirochaetes* (5.8%), and *Tenericutes* (3.1%) (Fig 1). The bacterial phyla *Firmicutes* and *Bacteroidetes* are known to dominate pig gastrointestinal tract [35]. The phylum composition observed in the present study is in agreement with that reported in several previous studies on pig manure slurry [12,13,36]. Out of the five most abundant phyla, only the relative abundance of *Bacteroidetes* varied significantly between seasons, having a higher relative abundance in the summer season (*P* < 0.05; Fig 1). Interestingly, in an anaerobic pig waste treatment lagoon, Cook et al. [37] found that the genus *Bacteroides*, which belongs to the phylum *Bacteroidetes*, had a very strong seasonal pattern; however, in contrast to our findings, there were more *Bacteroides* sp. in the winter season.

**Fig 2:**
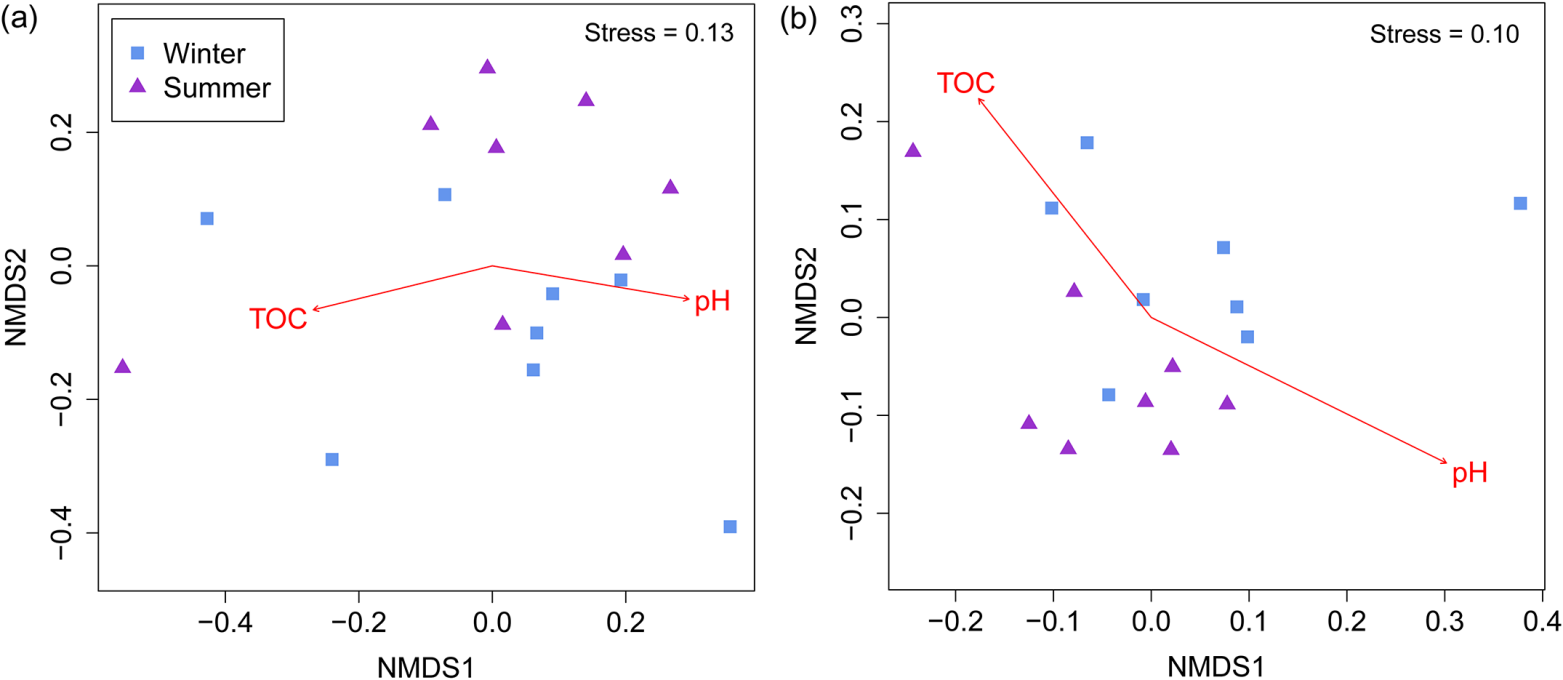
Nonmetric multidimensional scaling plot of bacterial (a) taxonomic (Bray-Curtis-based) and (b) phylogenetic (weighted UniFrac-based) community composition in pig manure slurry during the winter and summer seasons.

The ANOSIM test suggests that the bacterial taxonomic (Bray-Curtis-based) composition of pig manure slurry was not influenced by seasonal variations (ANOSIM *R* = 0.09, *P* = 0.12; Fig 2a); this non-significant relationship between season and taxonomic composition was consistent over different OTU cutoff levels (all *P* > 0.10; S1 Fig). However, the phylogenetic community composition had a weak, though significant, effect from the season (ANOSIM *R* = 0.20, *P* = 0.01; Fig 2b). It is unclear what mechanisms might result in significant seasonal variations in phylogenetic community composition; however it is possible that communities with similar taxonomic composition may differ in their phylogenetic community structure, and vice versa. Removing singleton OTUs did not alter the pattern in taxonomic (ANOSIM *R* = 0.09, *P* = 0.12) and phylogenetic community composition (ANOSIM *R* = 0.19, *P* = 0.02). The seasonal variability in the bacterial community composition was also reported earlier in pig waste treatment lagoons [37,38]. Next, we investigated the effect of physico-chemical variables on bacterial community composition by fitting the vectors of the physico-chemical variables onto the ordination space. In a recent study by Ducey and Hund [13], the use of the NGS method revealed that TKN, COD, ORP, total suspended solid, and DO were the major physico-chemical variables that influenced the bacterial community structure in pig wastewater anaerobic lagoons. However, in this study, the patterns of both the phylogenetic and taxonomic composition were strongly correlated with pH (taxonomic: *r* ^*2*^ = 0.78, *P* < 0.001; phylogenetic: *r* ^*2*^ = 0.85, *P* < 0.001) and TOC (taxonomic: *r* ^*2*^ = 0.67, *P* < 0.01; phylogenetic: *r* ^*2*^ = 0.61, *P* < 0.01). The fundamental role of pH in shaping the soil bacterial community composition is well known [39–41]; however, to the best of our knowledge, no study has illustrated the relationship between pH and bacterial community structure in pig manure slurry.

**Fig 3:**
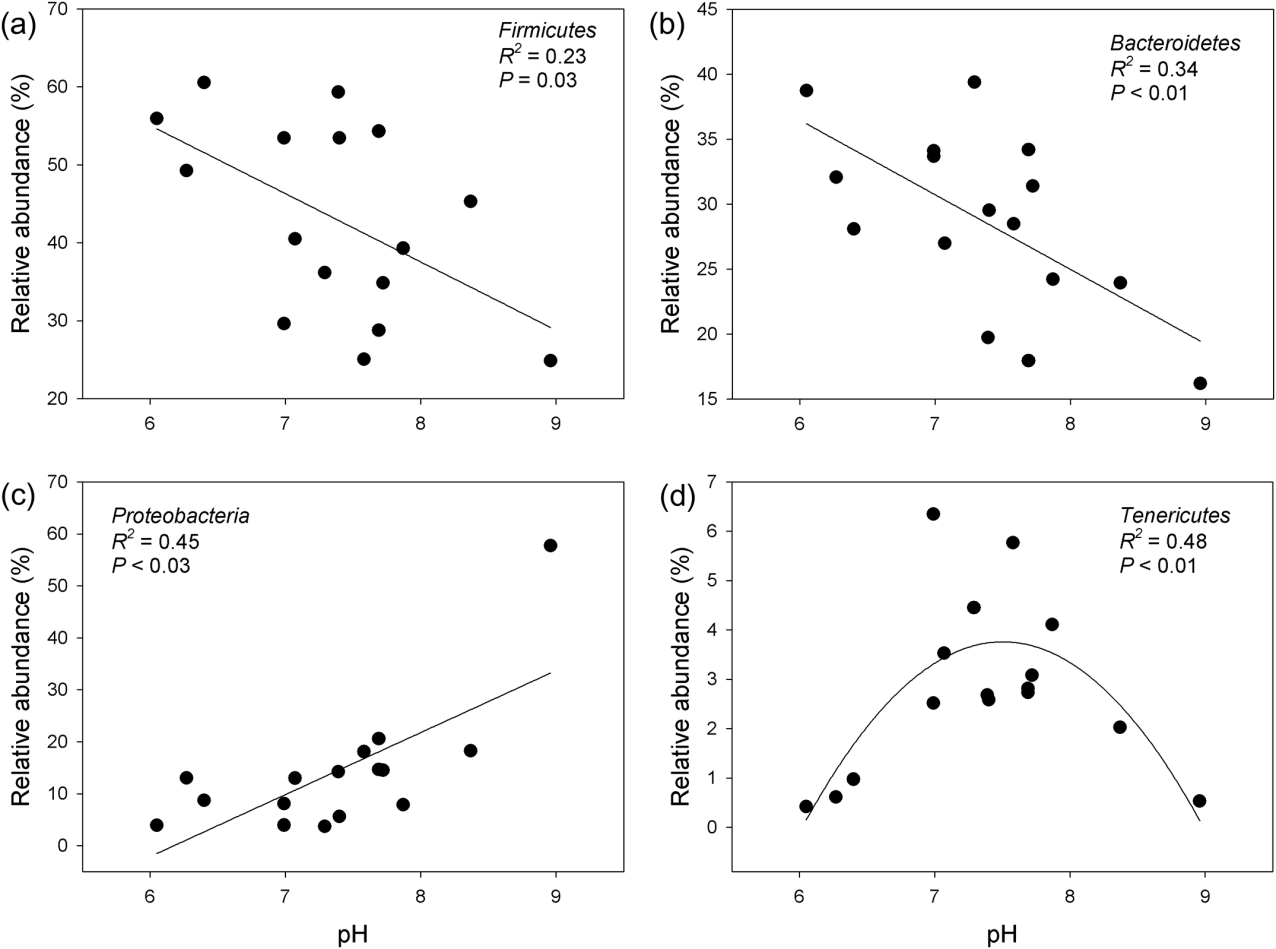
Relationship between the relative abundance of dominant bacterial phyla and pH. A linear model fit was the best fit for the relative abundance of all phyla except (d) *Tenericutes*, whose best fit was a quadratic model.

**Fig 4:**
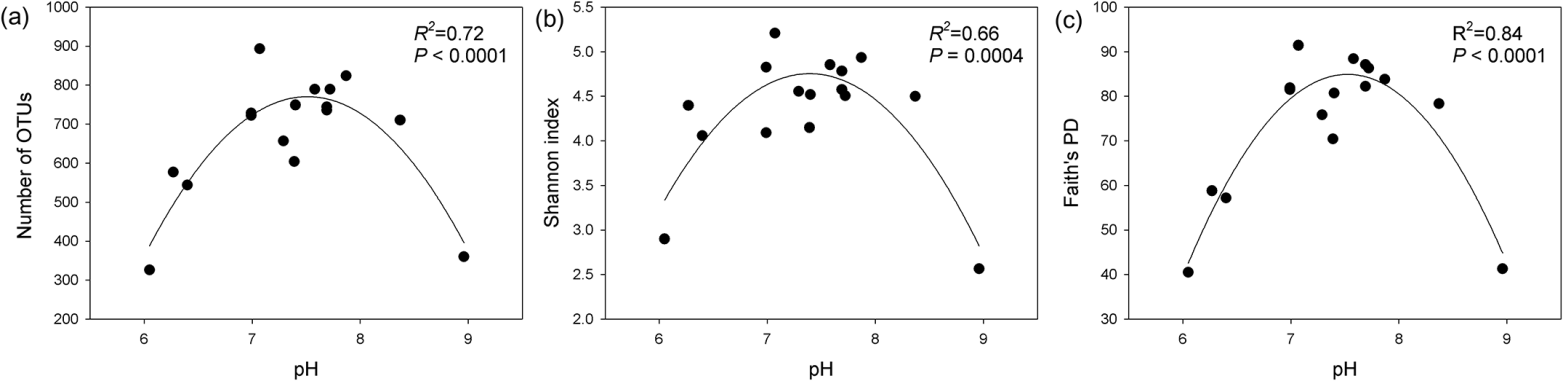
Relationship between pH and OTU richness (a), Shannon index (b), and Faith’s PD (c) of the bacterial community in pig manure slurry.

Slurry pH was the best predictor of the relative abundance of all dominant phyla, except for *Spirochaetes* (Fig 3). The relative abundance of *Firmicutes* and *Bacteroidetes* was negatively correlated with pH (Fig 3a and 3b), while *Proteobacteria* had a positive correlation with pH (Fig 3c), and *Tenericutes* had a unimodal relationship with pH (Fig 3d). The shifts in the relative abundance of dominant bacterial phyla across a pH gradient were observed in several previous studies on soils [40–42]; however, similar relationships were not previously observed in pig manure slurry. Bacterial OTU richness did not differ between seasons (*P* = 0.42). Similarly, none of the diversity indices differed between seasons (Shannon index *P* = 0.28; Faith’s PD *P* = 0.19). Both the OTU richness and diversity indices had a strong unimodal relationship with pH (OTU richness: *R* ^*2*^ = 0.72; Shannon index: *R* ^*2*^ = 0.66; Faith’s PD: *R* ^*2*^ = 0.84; all *P* < 0.05; Fig 4). Removal of singleton OTUs did not change this unimodal relationship between pH and various diversity indices (S2 Fig). Similar relationships were previously observed between pH and various diversity indices in various habitats [21,40,41]. These unimodal relationships between pH and diversity were previously explained by the fact that acidic and alkaline pH place strong physiological stress on bacteria and restrict the growth of bacterial populations, reducing diversity. Additionally, pH influences various other factors, such as nutrient availability [43], metal solubility and toxicity [44,45], which are important for maintaining diversity. The bacterial community composition and diversity observed in this study were highly heterogeneous across the samples; however, interestingly, they could easily be predicted by a single parameter, pH.

**Fig 5:**
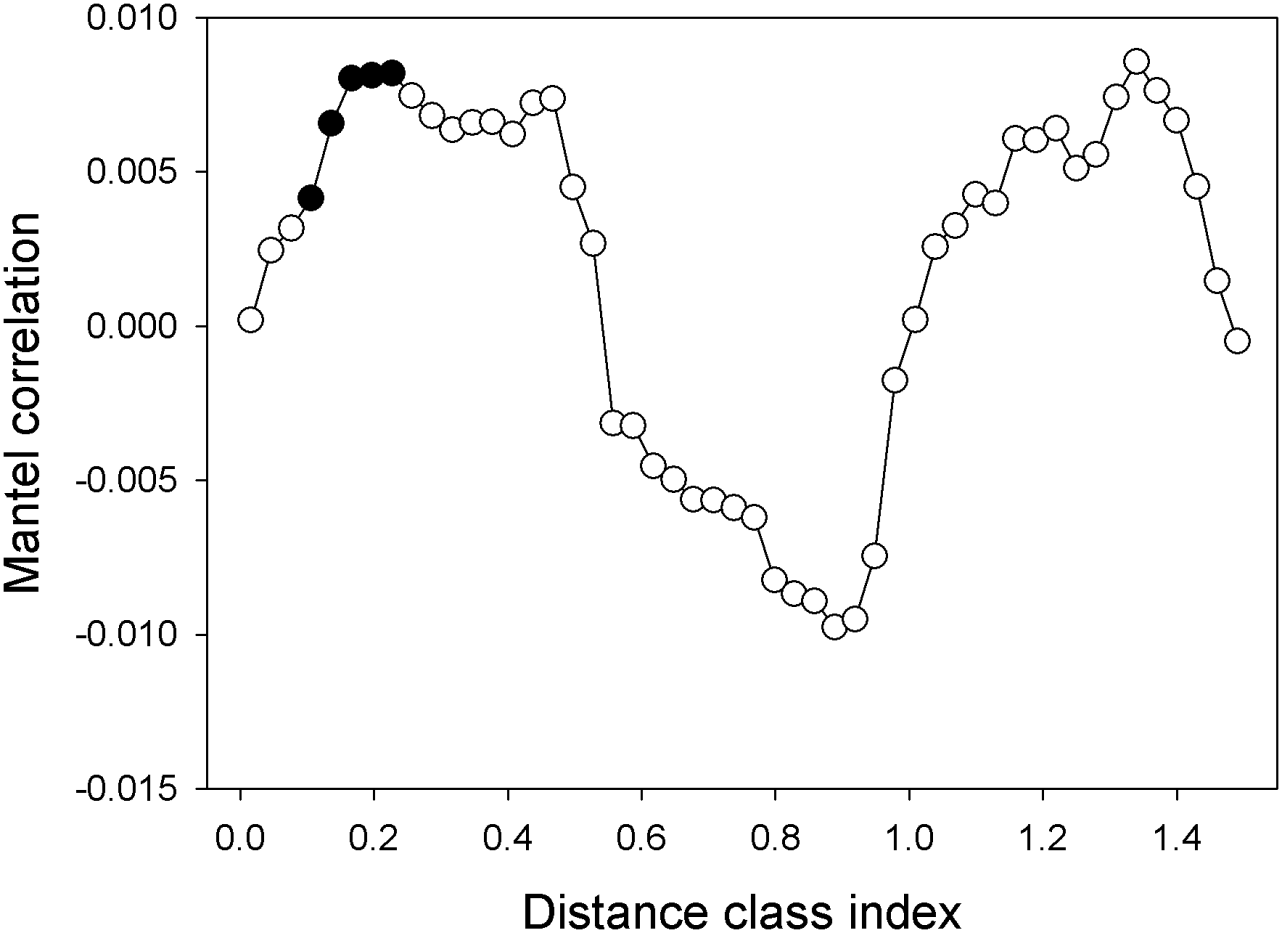
The Mantel correlogram showing phylogenetic signals (*P* < 0.01, filled squares) resulting from the correlation between OTU niche distances and OTU phylogenetic distances.

Phylogenetic signals are useful for inferring ecological processes [46,47]. A Mantel correlogram showed significant positive correlations over short phylogenetic distances (~ 15% of the maximum phylogenetic distance; Fig 5), suggesting closely related taxa are ecologically similar (i.e., closely related taxa tend to occur in the same environment). Our results are in agreement with several recent studies that were performed in various habitats [20,21,33]. To further analyze the closely related taxa, we calculated the SES.MNTD. The SES.MNTD deviated significantly from the expected value of zero, and the mean values were less than zero (mean = -7.3, *P* < 0.001), suggesting niche-based processes are more important than neutral processes in the community assembly of bacteria in pig manure slurry. Additionally, bacterial communities are more phylogenetically clustered than expected by chance.

**Fig 6:**
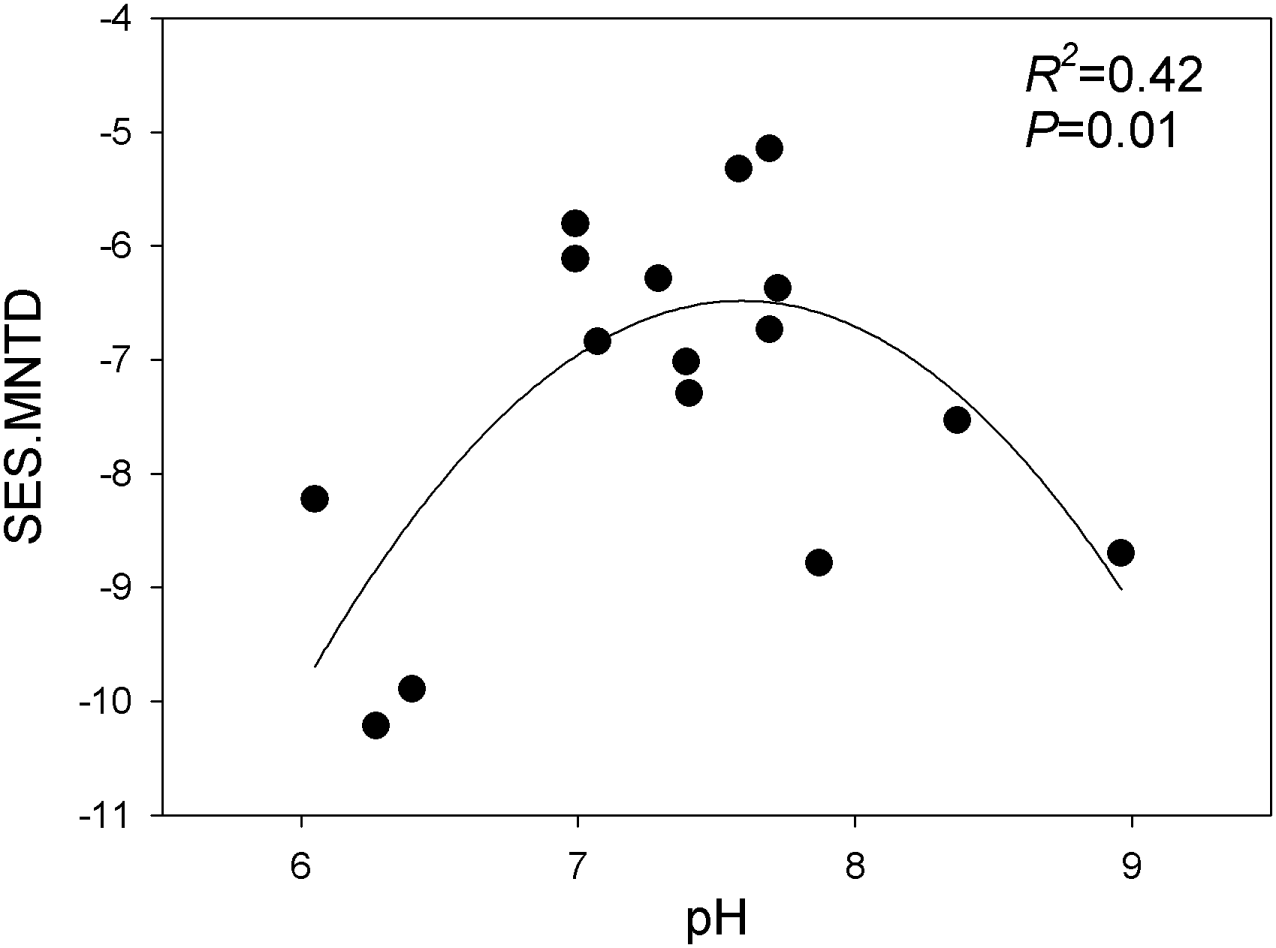
Relationship between pH and the standardized effect sizes of the mean nearest taxon distance (SES.MNTD).

As for the other diversity indices, the SES.MNTD had a strong unimodal pattern along pH (Fig 6), suggesting that in both acidic and alkaline pH range samples, closely related OTUs are more phylogenetically clustered. One of the possible explanations for this observation is that very few bacterial taxa can adapt and reproduce in acidic and alkaline habitats, resulting in environmental filtering from pH, which in turn increases the phylogenetic clustering in the sample present in acidic and alkaline pH ranges. The samples close to a neutral pH range were less phylogenetically clustered, indicating that neutral process are also important in the community assembly of closely related OTUs in these environments.

## Conclusions

In conclusion, our results indicate that pH emerges as the best predictor of the community composition and diversity of bacteria in pig manure slurry, while seasonal variations only influence the phylogenetic community composition. Significant phylogenetic signals were detected across short phylogenetic distances, revealing that bacterial OTUs are ecologically similar in pig manure slurry. Although, across all samples, a niche-based process primarily governed bacterial community assembly, neutral processes were also important in the samples close to neutral pH. These findings provided insight into the bacterial community structure and diversity of pig manure slurry as well as identify the processes that shape the community assembly of bacteria in pig manure slurry.

## Supporting Information

S1 Fig NMDS plot of bacterial community composition at 0.01 and 0.05 OTU cutoff level. (TIF)

S2 Fig Relationship between pH and OTU richness (a), Shannon index (b), Faith’s PD (c), and SES.MNTD. (TIF)
