# Supplementary figures and images for: Assessment of Bacterial Community Assembly Patterns and Processes in Pig Manure Slurry
Source: PLoS One. 2015 Sep 30;10(9):e0139437. doi: 10.1371/journal.pone.0139437 (PMC4589287; doi:10.1371/journal.pone.0139437)

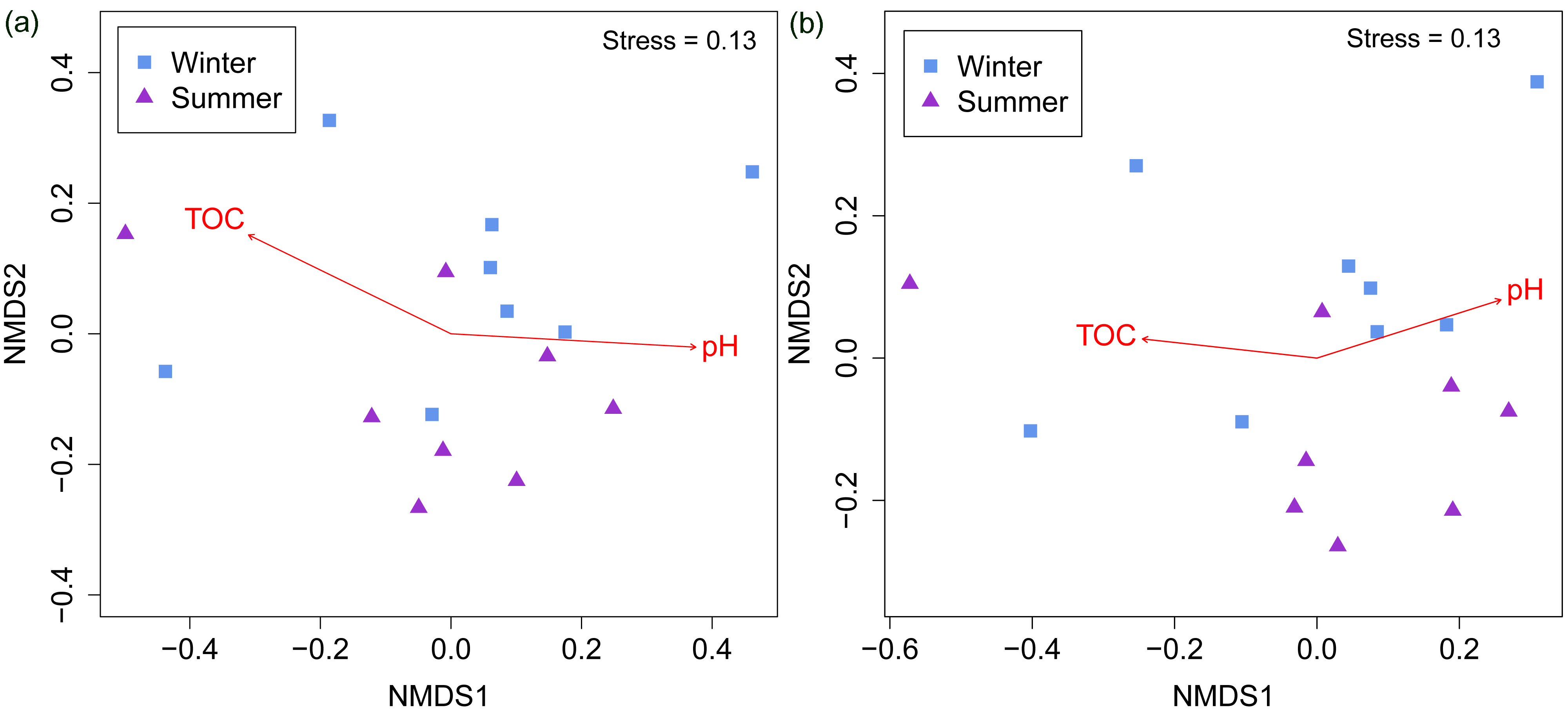

Supplement: S1 Fig — (TIF) [file pone.0139437.s001.tif]

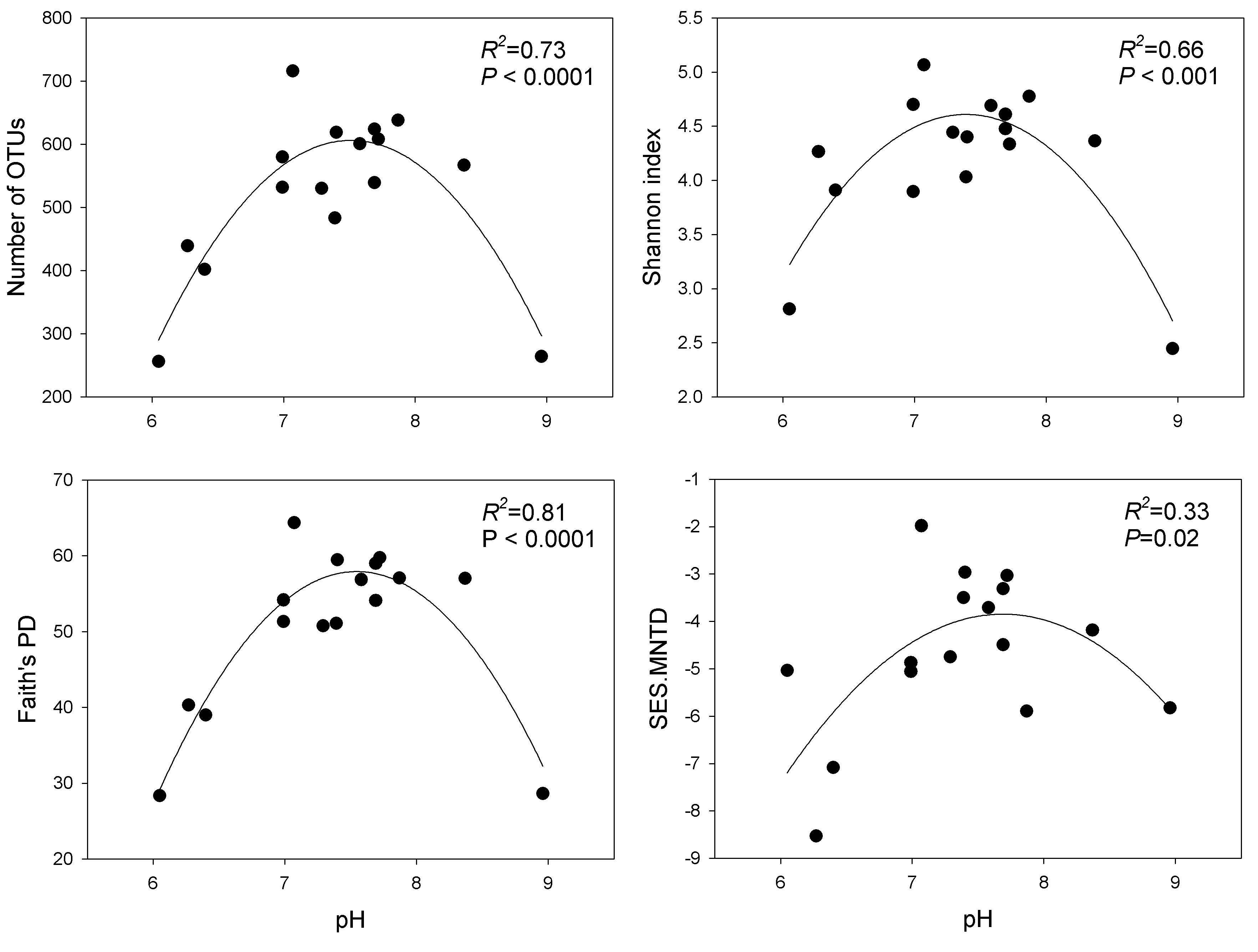

Supplement: S2 Fig — (TIF) [file pone.0139437.s002.tif]
